# Quercetin Reduced and Stabilized Gold Nanoparticle/Al^3+^: A Rapid, Sensitive Optical Detection Nanoplatform for Fluoride Ion

**DOI:** 10.3390/nano14231967

**Published:** 2024-12-07

**Authors:** Titilope John Jayeoye, Roselina Panghiyangani, Sudarshan Singh, Nongnuj Muangsin

**Affiliations:** 1 Department of Chemistry, Faculty of Science, Chulalongkorn University, Bangkok 10330, Thailand; 2 Department of Biomedic, Faculty of Medicine, Universitas Lambung Mangkurat, Kota Banjarmasin 70123, Indonesia; rpanghiyangani@ulm.ac.id; 3 Faculty of Pharmacy, Chiang Mai University, Chiang Mai 50200, Thailand; sudarshan.s@cmu.ac.th; 4 Office of Research Administration, Chiang Mai University, Chiang Mai 50200, Thailand

**Keywords:** quercetin, gold nanoparticles, aggregation agent, colorimetry, fluoride ion detection, real samples

## Abstract

In this contribution, facile synthesis of gold nanoparticles (AuNPs) at ambient conditions is reported based on the use of the polyphenolic compound quercetin (QT) as the reducing and stabilizing agent at room temperature (RT). Under alkali-induced pH adjustment of QT solution and stirring conditions at RT, QT could quickly reduce gold salt (Au^3+^) into its nanoparticle form (Au^0^), resulting in the formation of a sparkling red color colloidal solution (AuNPs) with an absorption maximum at 520 nm. Further, Fourier transform infrared spectroscopy (FTIR) was employed to showcase the role of QT in the nanomaterial’s synthesis process. The formed QT-AuNPs responded swiftly to Al^3+^ charging with color perturbation from red to grayish-purple, coupled with an absorption spectra red shift, owing to Al^3+^-induced aggregation of QT-AuNPs. However, when fluoride ion (F^−^) was pre-mixed with an optimized Al^3+^ concentration, reversed color changes from grayish-purple to red were observed with a blue shift in the absorption spectra. Simply put, F^−^ formed a complex with Al^3+^, thus preventing Al^3+^-induced aggregation of QT-AuNPs. The analytical response A_520_/A_650_ was linear with F^−^ concentration ranging from 25.0 to 250.0 µM and 250.0–600.0 µM, with a detection limit of 7.5 µM. The developed QT-AuNPs/Al^3+^ detection probe was selective to only F^−^ charging, in comparison with other possible interfering anions. Real sample potentiality of the developed sensor was demonstrated on tap water samples, toothpaste, and fluoride-rich mouthwash, with reliable accuracy.

## 1. Introduction

Anion detection has attracted huge interest in the scientific community owing to their roles in the human biochemical systems and the possibility of posing huge environmental challenges in cases of excessive exposure. Fluoride ion (F^−^) is one of the most important anions of interest, in view of its “sweet-sad” roles. In fact, F^−^ is needed for the development of healthy teeth by preventing dental caries or enamel demineralization and strong bones in humans [1]. On the other hand, excessive F^−^ intake through water consumption, foods, and other accessories can induce detrimental health consequences on the human reproductive system [2], teeth, bone fluorosis, kidney damage by urolithiasis, and other internal organ damage [3]. It should be mentioned also that F^−^ can be non-degradable, with high mineralization potentials on tissues, thus constituting a notable environmental pollutant, often generated from coatings-related manufacturing, pharmaceuticals, and pesticides [4,5]. From the foregoing, the World Health Organization (WHO) and the United States Environmental Protection Agency (USEPA) set maximum limits of F^−^ in drinking water at 1.5 mg/mL (79.0 µM) and 4 mg/mL (211 µM), respectively [6,7].

Reported methods for F^−^ detection include ion chromatography [8,9], high-performance liquid chromatography, HPLC [10], and chemiluminescence-based methods [11]. Others include electrochemical-sensor-based assays [12,13], spectrophotometric analysis [14], fluorescence-based sensing devices [15,16,17], and colorimetric detection assays [18,19,20]. While most of the mentioned detection systems for F^−^ above are suitable for sensitive quantification of the analyte in diverse matrices, notable inherent challenges are peculiar to some of the methods. Chromatography-based methods are generally tedious and time-consuming, oftentimes requiring rigorous sample preparation steps and the need for an experienced analyst to handle the equipment. Moreover, with point-of-care (POC) analytical detection materials now in high demand, chromatography methods would be found wanting in such settings. Hence, nanomaterial-based sensing assays, popularized by their high extinction coefficients, stability, and facile preparation routes, have attracted enormous research interest, especially in the simple detection of pollutants [21].

Gold nanoparticles (AuNPs) are one of the common metal nanoparticles (MNPs) with wide-ranging applications arising from their high stability, biocompatibility, and high surface tunability by various chemical and biological ligands/recognition probes [22]. On interaction with electromagnetic radiation, AuNPs surface electrons are set into resonance oscillation, exhibiting a phenomenon known as surface plasmon resonance (SPR) or, when confined to the MNPs colloidal solution, as localized surface plasmon resonance (LSPR). At this stage, MNPs display unique color and maximum light absorption and scattering. Under different external agents charging, MNPs of silver, gold, and copper possessing characteristics of naked-eye colors can transition to another color arising from the impact of the added agents, which is accompanied by a change in the absorption spectra. Such changes form the basis of colorimetric detection systems using plasmonic MNPs. These features have been employed in the design of optical detection strategies for manifold analytes [23,24,25,26].

Quercetin (3,3′,4′,5,7-Pentahydroxyflavone), a popular member of the flavonoids group of plants polyphenolic compounds, is popular for its pharmacological activities and biological potentials. It has strong anti-oxidant, anti-inflammatory, anti-bacterial, and anti-tumor properties [27]. Being a strong reducing agent, it has also been exploited for MNP synthesis, designated for biomedical applications [28,29]. Its other important features include strong chelating properties to metal ions like Fe^2+^, Cu^2+^, Fe^3+^, and Al^3+^ [30,31]. Since Al^3+^ can form a strong soluble complex with F^−^ [32], we thus conceptualized a detection strategy involving QT-derived AuNPs (QT-AuNPs), Al^3+^, and F^−^.

In this work, we report the detection of F^−^ in multiple matrices based on QT as the reductant/stabilizer of AuNPs at RT. The synthesized QT-AuNPs are highly stable, showing red color with an absorption maximum at 520 nm. Based on the strong affinity of Al^3+^ for QT, the synthesized QT-AuNPs could be easily aggregated under Al^3+^ charging, resulting in absorption maximum red shift and color perturbation. However, when Al^3+^ was premixed with F^−^, the aggregation potency of Al^3+^ on QT-AuNPs was suppressed in a concentration-dependent fashion, and a reversed absorption trend (blue shift) was observed. Thus, QT-AuNPs/Al^3+^ was investigated as an optical probe for facile, yet sensitive and selective detection of F^−^ in diverse real samples. To the best of our knowledge, this is the first report where QT-derived AuNPs were deployed for F^−^ detection in diverse real samples.

## 2. Materials and Methods

### 2.1. Materials and Chemicals

Quercetin and gold (III) chloride trihydrate HAuCl_4_·3H_2_O were procured from Sigma Aldrich (St. Louis, MO, USA). Sodium fluoride, NaF, and aluminum chloride hexahydrate (AlCl_3_·6H_2_O) were from Merck, Singapore. Other sodium and potassium salts deployed for interference studies include potassium bromide, KBr, from Carlo Erba reagents (Cornaredo, Italy); potassium chloride, KCl, and potassium iodide, KI, from UNIVAR Ajax Finchem Ltd. (Sydney, Australia); sodium nitrite, NaNO_2_, from RCI Labscan (Bangkok, Thailand); potassium thiocyanate, KSCN, potassium dihydrogen orthophosphate, KH_2_PO_4_, sodium nitrate, NaNO_3_, sodium sulfite, Na_2_SO_3_, sodium carbonate, Na_2_CO_3_, and sodium hydrogen carbonate, NaHCO_3_, were from LOBA Chemie, Mumbai, India; and sodium thiosulphate, Na_2_S_2_O_3_, sodium sulfate, Na_2_SO_4_, were from RCI Labscan, Thailand.

### 2.2. Instruments

Fourier transform infrared spectroscopy (FTIR) was recorded on a Nicolet iS50 FTIR spectrometer (Thermo Fisher Scientific, Waltham, MA, USA). The samples were ground with KBr into pellets and then inserted in the sample holder compartment, after which the spectra were collected. All absorption spectra were collected on a UV–Vis spectrophotometer, Agilent 8453 (Santa Clara, CA, USA), with a path length of 1 cm. Transmission electron microscopy (TEM) images of nanomaterials were collected on a JEOL JEM-2100 electron microscope, Tokyo, Japan. The sample solutions were dropped on a copper grid, then kept in a desiccator to dry before mounting on the device. Hydrodynamic diameters (Dh) were recorded on a dynamic light scattering (DLS) system, Malvern ZETASIZER NANO (Almelo, The Netherlands). Colloidal samples were measured into sample capillary cells and then inserted into the sample holder section of the equipment. Samples were run in triplicates (n = 100) for each run. All solutions were prepared using ultra-pure or Milli-Q water, (resistivity 18.2 MΩ·cm) obtained from a Millipore water purification system (Darmstadt, Germany). Photo images were collected using a Samsung smartphone (Suwon-si, Republic of Korea).

### 2.3. Preparation of QT-AuNPs

QT-AuNPs was prepared as follows. Into a 50 mL beaker, containing a magnetic stirrer, on a hot plate, 25 mL of water was added into the beaker under stirring, then 2 mL of 5 mM QT in ethanol was added, followed by 680 µL of sodium hydroxide (NaOH) 0.1 M solution, all at RT. Afterwards, 800 µL (optimized volume) of 20 mM HAuCl_4_.3H_2_O solution was injected under vigorous stirring. The synthesis reaction volume was maintained at 30 mL. The reaction mixture was allowed to proceed for 40 min, then the stirring was stopped, and the realized QT-AuNPs was transferred into an amber-colored bottle and then stored at 4 °C in the refrigerator before use.

### 2.4. Application of QT-AuNPs for F^−^ Detection

Colorimetric detection of F^−^ using QT-AuNPs was realized as follows. Into a 1.5 mL Eppendorf tube, 100 µL of prepared different concentrations of F^−^ was added to 100 µL of 1.5 mM Al^3+^ solution (Al^3+^ final concentration = 150 µM), then vortexed. 600 µL of water was added to the mixed reagents, then 200 µL of prepared QT-AuNPs was added. The total volume of the reaction system was 1 mL, and F^−^ final concentrations were varied from 0 to 600.0 µM. The detection mixture was vortexed, and the corresponding absorption spectra (in triplicates) or photo images were collected after 10 s.

### 2.5. Application of QT-AuNPs for F^−^ Detection in Real Samples

Practical applications of QT-AuNPs were demonstrated in F^−^ detection in diverse matrices, including tap water, toothpaste, and fluoride mouthwash. The samples were prepared as follows:

#### 2.5.1. Preparation of Tap Water for F^−^ Detection Based on QT-AuNPs/Al^3+^

This was realized following previous report [33]. Tap water was allowed to run for about 3 min, then collected in a beaker, covered with a glass plate, and was taken to the laboratory. The water was boiled (100 °C) to remove all chloride, then allowed to cool till it maintained RT. The prepared water sample and F^−^-spiked sample, at 100 and 400 µM, were charged on the QT-AuNPs/Al^3+^ probe. The realized absorbance values were converted to concentrations using the equation from the standard calibration plots, and the recovery was estimated using the equation

% Recovery = (C_a_ − C_b_)/ C_std_ × 100,

where C_a_ = Concentration of spiked sample; C_b_ = Concentration of unspiked sample; and C_std_ = Concentration of sample standard added.

#### 2.5.2. Preparation of Toothpaste for F^−^ Detection Based on QT-AuNPs/Al^3+^

Toothpaste was purchased from a shop in Bangkok, Thailand, and used. Shortly following the previous report [34], with modifications, 100 mg of toothpaste was weighed in a beaker, and then 10 mL of ultra-water was added. The mixture was sonicated for 20 min for complete dissolution of paste. The solution was centrifuged at 6000 rpm. and was further passed through a 0.22-micron membrane filter. The prepared solution was diluted five-fold to reduce sample matrix effect and then added to the QT-AuNPs/Al^3+^ system. The prepared toothpaste solution was also spiked with F^−^ standard at 100 and 400 µM, and the recovery percentage was calculated.

#### 2.5.3. Preparation of Fluoride Mouthwash for F^−^ Detection Based on QT-AuNPs/Al^3+^

Fluoride mouthwash was purchased from a pharmaceutical shop in Bangkok. Then, diluted twenty-fold in the laboratory with ultra-pure water. The diluted sample was charged on the QT-AuNPs/Al^3+^ detection system and further spiked with standard F^−^ at 50 and 200 µM. The sample recovery was estimated similarly to the description in Section 2.5.1.

## 3. Results and Discussion

### 3.1. Synthesis and Characterization of QT-AuNPs

**Scheme 1:**
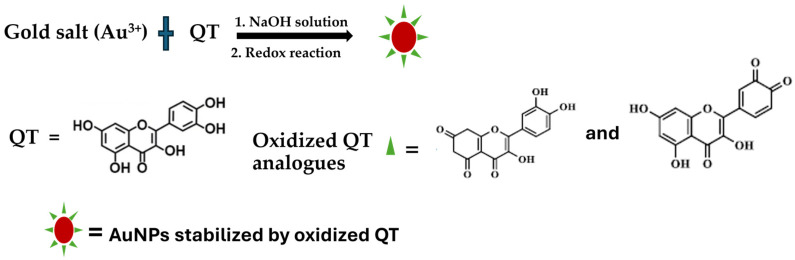
Synthesis strategy for QT-AuNPs at RT.

QT possesses multiple hydroxyl groups, which can act as a strong reductant towards gold salts (Au^3+^) as shown in Scheme 1. Under NaOH charging for pH adjustment, the multitudinous hydroxyl groups of QT can be deprotonated in solution, which increases its reductive efficiency and thus facilitates rapid nanoparticle nucleation, growth, and particle maturation processes [35]. Redox reaction between QT and gold salt results in the generation of colloidal AuNPs, stabilized by oxidized QT analog. As stated in Section 2.3, the starting materials mixing order includes QT, then NaOH for pH adjustment to 10, before gold salt addition. This created highly reproducible, fast, and well-formed AuNPs with a sparkling reddish color, showing an absorption maximum at 520 nm. The final pH of the synthesized QT-AuNPs is around 6.7.

The stabilizing or capping agents play significant roles in modulating the physico-chemical properties of the resulting nanomaterial. Thus, the presence of oxidized QT analogs on AuNP surfaces imbues the nanoparticles with Al^3+^-chelating surfaces, which helps in the facile and rapid aggregation of QT-AuNPs. Similar work has been investigated where tannic acid capping on AuNPs was exploited to control the redox activity and complexing with metal ions [36,37].

**Figure 1:**
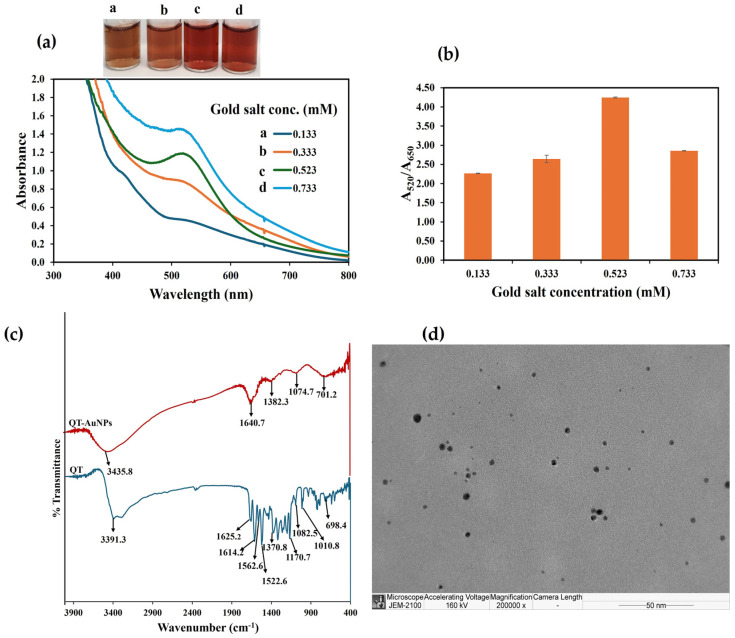
(**a**) UV–Vis absorption spectra of QT-AuNPs realized under different gold salt concentrations; the inset shows the phot images. (**b**) Plot of A_520_/A_650_ against gold salt concentrations. (**c**) FTIR of the QT-green line and QT-AuNPs-red line and (**d**) TEM image of QT-AuNPs produced under 0.523 mM gold salt concentration as the optimum condition.

The concentrations of gold salt in the synthesis reaction pot affect the QT-AuNPs formed. Consequently, this parameter was optimized. The addition of 200, 500, 800, and 1100 µL of 20 mM gold salt, in a 30 mL synthesis pot, produced final gold salt concentrations of 0.133, 0.333, 0.523, and 0.733 mM, as depicted in Figure 1a. As observed, as the gold salt concentration increased, the absorption intensity increased, while the peaks at 520 nm got sharper and less broadened. Moreover, the reddish color of AuNPs generated peaked at 0.523 mM (Figure 1a, inset). The absorbance ratio (A_520_/A_650_) could be used to assess the quality of the QT-AuNPs produced. As shown in Figure 1b, the ratio increased with the concentration of gold salt until 0.523 mM, then decreased afterwards. The higher the ratio, the better the absorption spectra of QT-AuNPs formed and the more reddish the AuNPs formed. From the foregoing, the synthesis of QT-AuNPs was carried out at 0.523 mM gold salt in the final reaction pot.

Materials characterization, especially through functional group changes observed in QT and QT-AuNPs, is depicted in Figure 1c. Notable peaks of QT (Figure 1c-blue line) include 3391.3 cm^−^^1^, which is assigned to the C-H stretching of OH groups; 1625.2 and 1614.2 are for C=O stretching absorption; peaks at 1562.6 and 1522.6 cm^−^^1^ are from the C=C aromatic ring stretching absorption; the 1370.8 cm^−^^1^ peak is from the bending OH groups from phenolic functional groups; and other peaks at 1170.7, 1082.5, and 1010.8 cm^−^^1^ are assigned to the C-H of aromatic compounds [38]. For QT-AuNPs (Figure 1c-red line), the observed peaks include 3435.8 cm^−^^1^, typical of C-H stretching of OH groups, and 1640.7, 1382.3, 1074.7, and 701.2 cm^−^^1^, which are similar to the peaks identified in pure QT. Generally, the peaks in QT-AuNPs shifted to a higher value, while the peak at 701.2 cm^−^^1^ is assigned to the Au-O bond present on the synthesized AuNPs surfaces [39]. This shows that the realized AuNPs are stabilized by an organic compound with a similar structure to the pure QT.

The TEM of QT-AuNPs (Figure 1d) shows highly dispersed particles, perfectly spherical-shaped materials with homogeneous sizes. Measuring the sizes of 50 particles on ImageJ software (version 1.54k) shows the particle sizes ranged between 1 and 20 nm, with an average size around 7.85 ± 1.2 nm (Figure S1). The stability of the synthesized QT-AuNPs when stored at 4 °C in the refrigerator is shown in Figure S2. As can be observed, the UV–Vis remained unchanged, which confirmed that the synthesis protocol is capable of generating AuNPs of impressive properties.

### 3.2. Application of QT-AuNPs in F^−^ Detection

**Figure 2:**
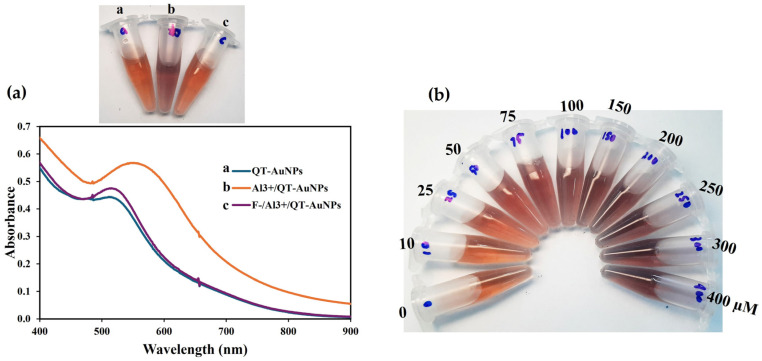
(**a**) UV–Vis absorption spectra of a—QT-AuNPs, b—Al^3+^ charging on QT-AuNPs, and c—Al^3+^/F^−^ pre-mixed before QT-AuNPs addition; Al^3+^ = 150 µM and F^−^ = 600 µM. (**b**) Photo images of varying concentrations of Al^3+^ (0–400 µM) on QT-AuNPs.

Colorimetric detection of F^−^ was designed using Al^3+^ as an aggregation agent of QT-AuNPs in an aqueous environment. The exceptional binding affinity of Al^3+^ to phenolic compounds has been reported in numerous works [40,41,42]. Thus, we hypothesized the possible aggregation of QT-AuNPs on Al^3+^ addition and the reversal of Al^3+^-induced aggregation on pre-mixing Al^3+^ and F^−^, owing to the higher affinity of Al^3+^ for F^−^ forming highly soluble aluminum fluoride [43]. This was summarily investigated as shown in Figure 2a. The absorption spectra of QT-AuNPs show a sharp peak at 520 nm (Figure 2a, line a), with a sparkling reddish color (Figure 2a, picture a). The addition of Al^3+^ resulted in an absorption spectra red shift (Figure 2a, line b), with a grayish-purple color formation (Figure 2a, picture b). Further, the absorption spectra broadened, arising from QT-AuNPs aggregation. However, on pre-mixing Al^3+^ with F^−^, a blue shift in absorption spectra was observed (Figure 2a, line c), with the formation of reddish color similar to QT-AuNPs (Figure 2a, picture c). This indeed validated the proposed hypothesis that QT-AuNPs/Al^3+^ could be deployed for colorimetric detection of F^−^. It must further be mentioned also that the aggregation of QT-AuNPs, induced by Al^3+^, does not follow the usual red to blue pattern; however, a naked-eye observable color perturbation can be perfectly identified, while the absorption spectra changes achieved can be skillfully tuned for sensitive F^−^ detection.

#### 3.2.1. Parameter Optimization for QT-AuNPs Towards F^−^ Detection

➢ *Effect of concentration of aggregation agent- Al^3+^*

As it is generally known, in an anti-aggregation-based detection strategy, the concentration of the aggregation agent is about the most important parameter affecting the analyte’s response. An excess of the aggregation agent will require a higher concentration of the analyte to impart an anti-aggregation effect and thus lead to poor sensitivity and a high limit of detection (LOD). The effect of increasing concentration of Al^3+^ ion on QT-AuNPs is shown in Figure 2b. As revealed, as the concentration of Al^3+^ increased in the detection system, QT-AuNPs color perturbations from reddish to grayish-purple equally increased. As can be observed by the naked eye, Al^3+^ concentration at 150 µM is sufficient to make a distinctive color differentiation compared with the QT-AuNPs. Consequently, Al^3+^ concentration was set at 150 µM.

➢ *Aggregation kinetics of Al^3+^ on QT-AuNPs*

The aggregation kinetics of Al^3+^ on QT-AuNPs was studied using Al^3+^ concentration at 150 µM by collecting the absorption spectrum at regular time intervals. As shown in Figure S3, the aggregation effect of Al^3+^ on QT-AuNPs was very rapid as the absorption spectra stabilized quite quickly, while the plot of absorbance at 650 to 520 nm (A_650_/A_520_) against time shows a very rapid aggregation process after sample incubation at room temperature. Consequently, the absorption spectra and the photographic images in the work were collected 10 s after the samples were vortex mixed.

#### 3.2.2. Analytical Performance of QT-AuNPs/Al^3+^ Probe Towards F^−^ Detection

The analytical features of the QT-AuNPs/Al^3+^ detection probe towards F^−^ detection were investigated, with a view to presenting a facile and reliable F^−^ detection system that is comparable with reported works.

➢ *Detection sensitivity*

**Figure 3:**
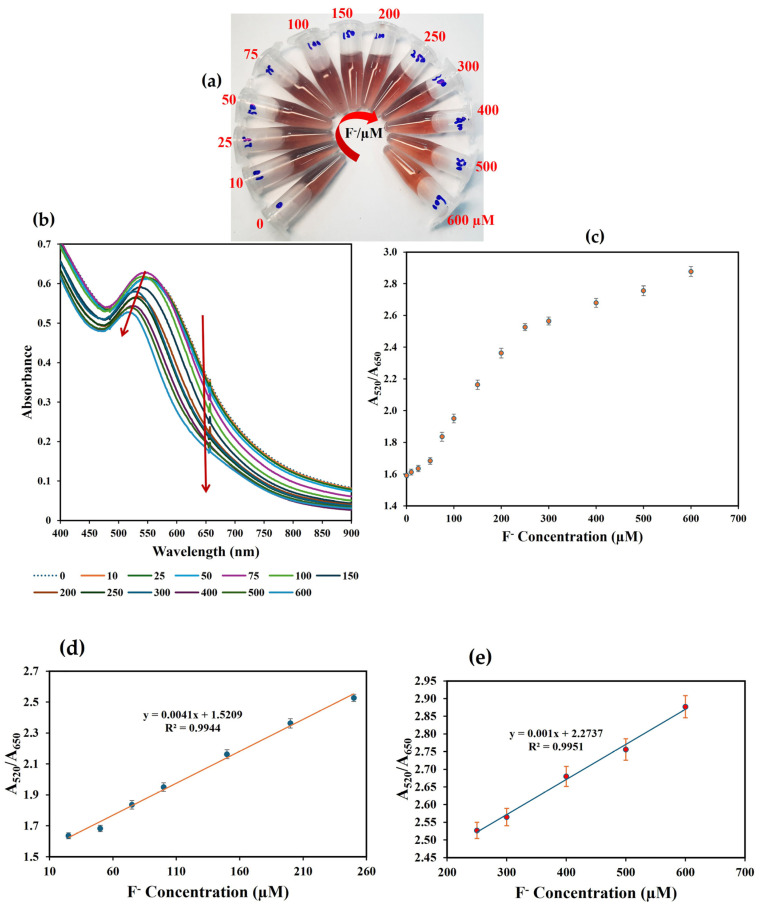
(**a**) Photo images of the QT-AuNPs/Al^3+^ colorimetric probe under F^−^ ion charging from 0 to 600 µM. (**b**) UV–Vis absorption spectra of QT-AuNPs/Al^3+^ detection probe corresponding to the photo images obtained in F^−^ detection from 0 to 600 µM. (**c**) Plot of (A_520_/A_650_) against F^−^ concentration from 0 to 600 µM. Plot of (A_520_/A_650_) against F^−^ concentration from (**d**) 25–250 µM, and (**e**) 250–600 µM. The concentration of Al^3+^ = 150 µM.

Sensitivity is an important analytical detection performance parameter, which measures the rate of response to a slight change in sample concentration. This is oftentimes carried out by charging an incremental concentration of the analyte on the detection probe, after which the analytical response is collected. As can be observed in Figure 3, the addition of increasing F^−^ concentrations to the QT-AuNPs/Al^3+^ system, under Al^3+^ charging at 150 µM, reversed color transitions from grayish-purple to red (Figure 3a), was observed. The absorption spectra, as revealed in Figure 3b, depicted progressive reduction in the absorbance at 650 nm and at 520 nm. The very broad absorbance of the QT-AuNPs/Al^3+^ probe, with absorption maximum at a longer wavelength, blue-shifted consistently towards 520 nm, while the absorption at 650 nm decreased over the full F^−^ concentration range. It is important to state that at 650 nm, the absorption spectra are better spaced out without much interlay, which is more beneficial for reproducible and consistent analytical parameters estimation. It ensures that better linear ranges are obtained at lower concentrations of the analytes in comparison to other wavelengths, such as 700 and 800 nm. Thus, the analytical response (A_520_/A_650_), plotted against the full F^−^ concentration range (0–600 µM), confirmed the progressive increment in the ratio (Figure 3c). It should be mentioned that the higher the ratio, the higher the reddish color of the generated sample, while low values of the ratio are attributed to grayish-purple color or higher aggregation of AuNPs. F^−^ concentrations over the full concentration range showed two linear ranges from 25 to 250 µM (Figure 3d), with the calibration plot equation A_520_/A_650_) = 0.0041[F^−^] + 1.5209, with an R^2^ value at 0.9944, and from 250 to 600 µM (Figure 3e), with a calibration plot equation A_520_/A_650_) = 0.001[F^−^] + 2.2737, with an R^2^ value at 0.9951. The detection limit is estimated using the equation 3.3 × (α/S), where α is the standard deviation of blank (QT-AuNPs/Al^3+^), for n = 10, and S is the slope of the calibration plots. Accordingly, the calculated LOD for the QT-AuNPs/Al^3+^ detection probe is 7.5 µM. The obtained LOD is far below the acceptable limits of F^−^ in drinking water at 79 and 211 µM from WHO and USEPA, respectively. It is important to add also that theoretically, one mole of Al^3+^ required 3 moles of F^−^ to form the complex AlF_3_. Thus, Al^3+^ concentration at 150 µM should, by extension, be completely consumed by F^−^ concentration at about 450 µM. In this work, the concentration of Al^3+^ was completely neutralized by F^−^ concentration at 600 µM (Figure 3a–c). This can be explained by considering that the properties of nanomaterials are not exactly the same as those shown at the atomic, molecular level, or bulk precursors [44]. The reaction surrounding materials, such as water, temperature, humidity, etc., may affect detection responses. This similar trend was observed in a related work [45].

The obtained LOD and the detection linear ranges were compared with reported works for F^−^ detection in diverse matrices, as presented in Table 1 [46,47].

As detailed, the present detection probe shows comparable analytic features with reported works for F^−^ detection. As can be observed, the LOD of the QT-AuNPs/Al^3+^ system at 7.5 µM is lower than previous AuNPs-based sensors [18,20], while its wide linear detection range is far better than a few of the reported nanomaterials-based colorimetric detection methods [18,20]. The wide linear ranges achieved in the present work will be of much interest in real sample detection, and thus the QT-AuNPs/Al^3+^ system would present a robust and practical F^−^ detection strategy in multiple sample matrices. In comparison to the fluorescence-based assays [48,49,50,51], the use of a simple spectrophotometer for data acquisition in the present study is an added advantage over fluorimetry, as the possibility of auto-fluorescence of samples could hamper its utilitarian application for F^−^ detection in real samples. Moreover, though the electrochemical systems often present low LODs in analytical detections, owing to the improved sensitivity of carefully designed electrodes, they require potentiostats and other electrochemical workstations; hence, they may not be easily accessible to researchers, especially those in economically challenged environments [52].

➢ *Mechanism of F^−^ detection based on QT-AuNPs/Al^3+^*

**Scheme 2:**
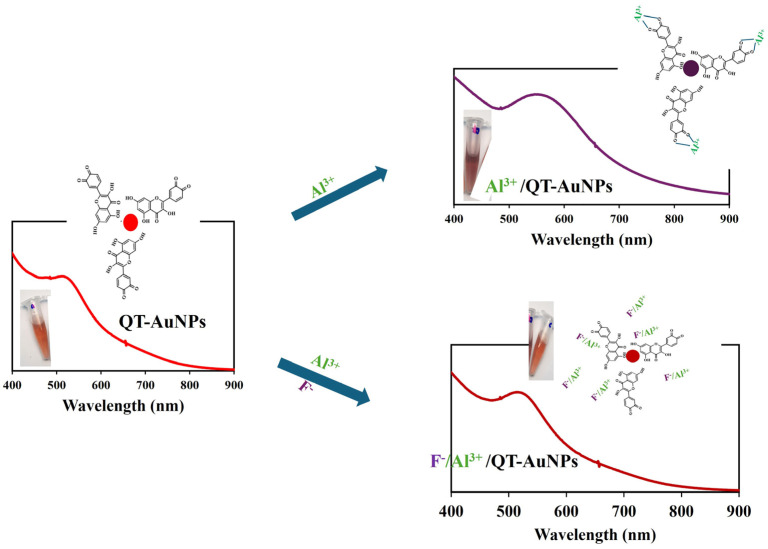
Mechanism of the QT-AuNPs/Al^3+^-based detection system for F^−^.

The possible detection mechanism of F^−^ detection based on the QT-AuNPs/Al^3+^ probe is explained below, as shown in Scheme 2. AuNPs were synthesized using QT as the reductant/stabilizer; under redox reaction, this leads to the generation of reddish AuNPs stabilized by oxidized QT analog as explained previously in Scheme 1. The addition of Al^3+^ to reddish QT-AuNPs, having a sharp absorption peak at 520 nm, led to the AuNPs aggregation, based on Al^3+^ chelation to the free diketone form of QT available on the AuNPs surfaces [53]. Consequently, the sparkling reddish color of QT-AuNPs changed to grayish-purple color (Scheme 2-sample Al^3+^/QT-AuNPs inset), signifying particle aggregation, with concomitant absorption peak broadening and red shift (Scheme 2-sample Al^3+^/QT-AuNPs UV–Vis). On pre-mixing Al^3+^ and F^−^, before QT-AuNPs injection, owing to the stronger complex formation between Al^3+^ and F^−^, highly soluble aluminum fluoride is formed, which limits the aggregation influence of Al^3+^ on QT-AuNPs. As a result, the reddish color of QT-AuNPs is restored (Scheme 2-sample F^−^/Al^3+^/QT-AuNPs inset), and a blue shift in the absorption spectrum is furnished (Scheme 2-sample F^−^/Al^3+^/QT-AuNPs UV–Vis). To further buttress the aggregation of QT-AuNPs in the presence of Al^3+^, the functional group changes were profiled by obtaining the FTIR spectrum. As shown in Figure S5. Few peaks prominent at 3410.8, 1635.3, and 1015.5 cm^−1^ were identified, which are assigned to the C-H stretching of the OH group and C=O of ketone (1635.3 cm^−1^), which all experienced significant shifts when compared to QT-AuNPs (Figure 1c) above, and the notable disappearance of major peaks between 1400 and 700 cm^−1^ (marked in red, Figure S5) shows QT-AuNPs had undergone prominent changes on Al^3+^ charging. Thus, Al^3+^ chelation to the free and available binding sites on QT-AuNPs engenders rapid aggregation of initially sparkling reddish color QT-AuNPs to grayish-purple.

**Figure 4:**
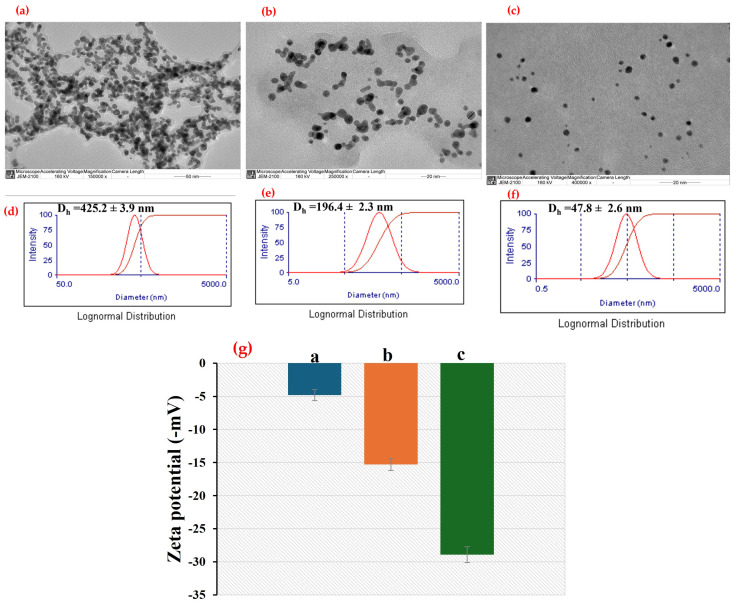
(**a***–***c**) TEM images of the QT-AuNPs/Al^3+^ colorimetric probe under F^−^ ion charging at 0, 150, and 500 µM. (**d***–***f**) Hydrodynamic diameter from DLS corresponding to (**a***–***c**) of TEM and (**g**) zeta potential under F^−^ concentration charging at a = 0, b = 150, and c = 500 μM. The concentration of Al^3+^ = 150 µM.

The nanoparticle changes on Al^3+^ treatment and F^−^-induced reversal of QT-AuNPs aggregation were also supported with TEM, hydrodynamic diameter acquisition on a DLS machine, and zeta potential profiling as shown in Figure 4. F^−^ concentrations at 0 (blank sample or Al^3+^ /QT-AuNPs), 150, and 500 μM are provided. An intense particle aggregation of QT-AuNPs/Al^3+^ probe, in the absence of F^−^ ion (i.e., F^−^ at 0 μM), is shown in Figure 4a, and the associated average particle size from TEM was estimated as 8.69 ± 2.1 nm. Less aggregation was observed when F^−^ was fixed at 150 µM (Figure 4b), with an average particle size of 5.75 ± 1.5 nm and highly dispersed particles, when F^−^ concentration was at 500 µM (Figure 4c), with a calculated average particle size of 2.66 ± 0.7 nm. The hydrodynamic diameters (D_h_) for F^−^ addition at 0, 150, and 500 µM are shown in Figure 4d–f, with D_h_ values at 425.2 ± 3.9 nm, 196.4 ± 2.3 nm, and 47.8 ± 2.6 nm, respectively. This shows the particles clumpiness decreases as F^−^ concentration increases, owing to the reduction in the aggregation capacity of Al^3+^ on QT-AuNPs in the presence of the analyte. The particle size estimated from TEM is much different from that obtained from the DLS machine, simply because TEM works with dried samples, while particle size based on DLS is conducted in an aqueous environment. Consequently, the hydration shell of water is estimated with the actual sample particle size, hence the increased size estimates common in DLS [54]. The zeta potential equally followed a similar trend as shown in Figure 4g. The zeta potential of QT-AuNPs/Al^3+^ probe in the presence of F^−^ ion at 0, 150, and 500 µM showed values of −4.8 ± 0.9, −15.3 ± 0.9, and −28.9 ± 1.2 mV, respectively. This signifies the particles stability increased as F^−^ concentrations were increased from 0 to 500 µM. Generally, the zeta potential of highly stable colloidal nanoparticles has been proven to fall around ±30 mV [55].

➢ *Precision and Selectivity*

**Figure 5:**
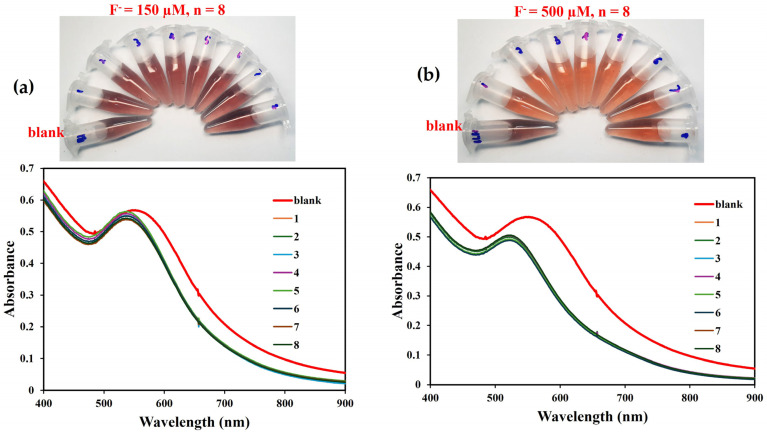

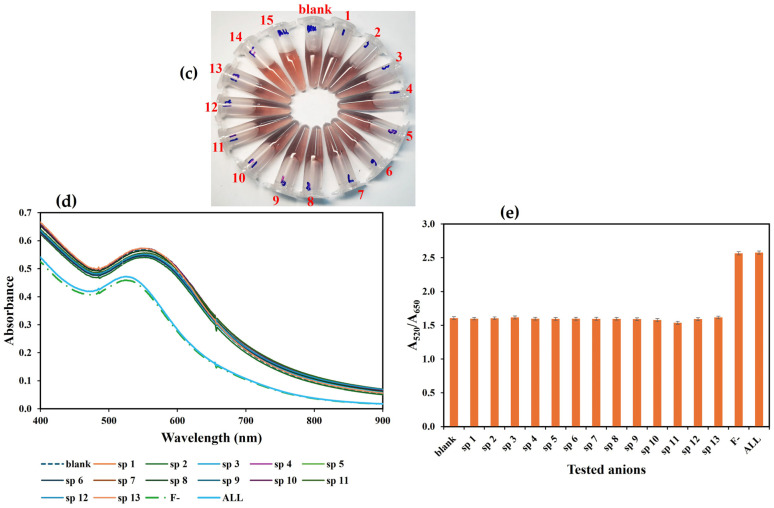
UV–Vis absorption spectra of QT-AuNPs/Al^3+^ for repeated F^−^ detection at (**a**) 150 and (**b**) 500 µM; the inset shows the photo images of the blank and the tested F^−^, for n = 8. (**c**) Photo images of F^−^ and other possible interfering anions, where 1 = Br^−^, 2 = Cl^−^, 3 = I^−^, 4 = NO_2_^−^, 5 = NO_3_^−^, 6 = SO_4_*^2−^*, 7 = S_2_O_3_^−^, 8 = SO_3_*^2−^*, 9 = HCO_3_^−^, 10 = CO_3_*^2−^*, 11 = SCN^−^, 12 = H_2_PO_4_^−^, 13 = HPO_4_*^2−^*, 14 = F, 15 = ALL, F^−^ = 300 µM, and the others were 3000 µM. The concentration of Al^3+^ = 150 µM, (**d**) UV–Vis absorption spectra corresponding to the shown images in (**c**,**e**). Plot of (A_520_/A_650_) against tested anions and F^−^.

The precision of the QT-AuNPs/Al^3+^ probe towards F^−^ detection was evaluated by charging F^−^ at low and high concentration ranges from the calibration plot. Precision is oftentimes expressed as reproducibility and repeatability, using the relative standard deviation (RSD%). It connotes the ability of a detection system to be repeated without a significant deviation. For intra-day precision, F^−^ at 150 and 500 µM was charged on the QT-AuNPs/Al^3+^ probe (n = 8) and compared with the blank, as shown in Figure 5. The absorption spectra experienced no significant changes for 150 and 500 µM F^−^ concentrations (Figure 5a,b), while similar color images were obtained accordingly (Figure 5a,b, insets). The RSD (standard deviation/mean × 100) was acquired, and values of 1.7 and 2.2% were obtained for the tested concentrations. The tested concentrations of F^−^ were also charged on the probe for three consecutive days to investigate the inter-day precision, where values of 2.1 and 2.7% were estimated for F^−^ at 150 and 500 µM, respectively. These show that the QT-AuNPs/Al^3+^ is reliable for F^−^ detection without notable response variability.

The selectivity of the QT-AuNPs/Al^3+^ probe towards F^−^ detection, in comparison with other possible interfering anions, was tested. Multiple anions, including Br^−^, Cl^−^, I^−^, NO_2_^−^, NO_3_^−^, SO_4_^2−^, S_2_O_3_^−^, SO_3_^2−^, HCO_3_^−^, CO_3_^2−^, SCN^−^, H_2_PO_4_^−^, HPO_4_^2−^, F^−^, and an additional F^−^ simulated real sample situation, comprising F^−^ and other anions mixed together (ALL), were charged on the probe, while F^−^ was maintained at 300 µM and interferents at ten-fold concentration (3.0 mM). As presented in Figure 5c, the addition of manifold interfering anions at ten times the concentration of F^−^ (1–13, Figure 5c) presented no observable color changes to the blank. While F^−^ and F^−^ mixed with interferants (ALL) show reddish color (Figure 5c). Furthermore, the absorption spectra of the blank and the tested species (Figure 5d) show the interfering anions are not widely different from the blank, while F^−^ at 300 µM and ALL exhibited different absorbances. This validated the exceptional preference of the QT-AuNPs/Al^3+^ probe towards F^−^ detection, such that the addition of other anions at ten-fold concentrations imparted no notable changes on the probe. The plot of absorbance ratios (A_520_/A_650_) against the tested species (Figure 5e) shows the value of other interfering anions (1–13) displayed responses not significantly different from the blank (*p* ≥ 0.05), while F^−^ and the simulated sample (ALL) show values that are much higher than the blank. These confirmed that the detection mechanism is selective and specific to F^−^ detection owing to the high complex formation affinity of Al^3+^ and F^−^.

#### 3.2.3. Real Sample Application of the QT-AuNPs/Al^3+^ Probe in F^−^ Detection

**Figure 6:**
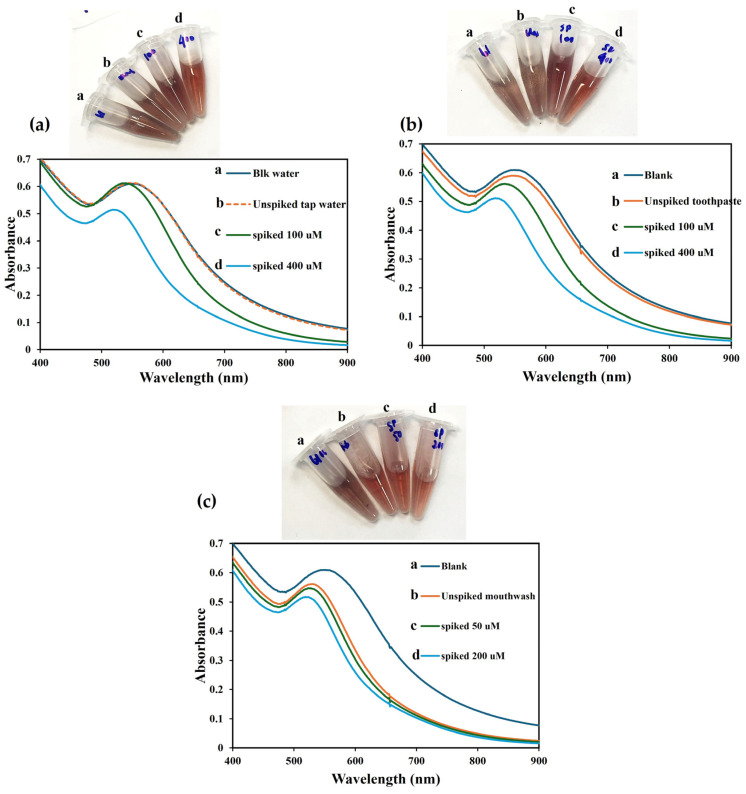
UV–Vis absorption spectra of QT-AuNPs/Al^3+^ for real samples F^−^ detection, including (**a**) tap water, (**b**) toothpaste, and (**c**) fluoride-rich mouthwash; the insets show the photo images corresponding to the blank sample, unpiked sample, and standard F^−^-spiked real samples at two concentrations, c and d. Al^3+^ concentration was at 150 µM.

The practical demonstration of the detection probe towards F^−^ detection in multiple real samples (tap water, toothpaste, and fluoride-rich mouthwash) was investigated using the standard addition method. The sample preparation steps have been detailed in the materials and methods section. Figure 6 shows the photo images and the absorption spectra of F^−^ detection, revealing the QT-AuNPs/Al^3+^ probe or blank, the real sample without standard F^−^ addition or unspiked sample, and the F^−^ samples spiked with standard F^−^ at two concentration ranges, or spiked samples. As can be seen, with the addition of a real sample to the QT-AuNPs/Al^3+^ probe, no color change was observed in the water sample compared to the blank (Figure 6a), only slight color change for toothpaste (Figure 6b), and significant color change for fluoride-rich mouthwash (Figure 6c). This shows the probe can respond to varying F^−^ concentrations in diverse matrices. The analytical response (A_520_/A_650_) was obtained from the UV–Vis absorption spectra of each sample in triplicate and was converted to the concentration values using the appropriate calibration plots, as presented in Figure 3d,e. Accordingly, the % recoveries were estimated (using the formula in Section 2.5.1), and the F^−^ concentrations in the different real samples are shown in Table 2. No F^−^ was detected in tap water, while F^−^ concentrations after the dilution factor for toothpaste and fluoride-rich mouthwash (5 and 20), respectively, were calculated as 356.5 ± 2.8 µM and 7955.6 ± 3.2 µM. That is, the obtained F^−^ concentrations from the real samples were multiplied by the corresponding dilution factors. The recoveries for standard F^−^-spiked samples ranged between 96 and 102%, attesting to the high efficacy and accuracy of the probe towards F^−^ detection.

## 4. Conclusions

In this work, QT-AuNPs were synthesized at ambient conditions (room temperature-RT), and was applied for F^−^ ion detection in aqueous environment. The QT-AuNPs is highly stable for over two months when kept in the refrigerator, showing reddish color with an absorption maximum at 520 nm. The addition of Al^3+^ could easily induce an aggregation effect on QT-AuNPs, which is marked by naked-eye observable color change and an absorption maximum red shift. This was proved to be attributed to the chelation effect of Al^3+^ on the free diketones on the QT structure serving as a stabilizing agent for QT-AuNPs. However, when our analyte F^−^ was pre-mixed with Al^3+^, reversed color and absorption spectra transitions were easily observed and were deployed for F^−^ detection. The detection probe QT-AuNPs/Al^3+^ shows impressive precision and selectivity, even in the presence of possible interferents at ten-fold higher concentrations of F^−^, and reliable real sample effectivity for F^−^ profiling in tap water, toothpaste, and fluoride-rich mouthwash.

## Figures and Tables

**Table 1:** Comparison of the QT-AuNPs/Al^3+^ probe with reported detection methods for F^−^.

Detection Probe	Analytical Method	Linear Range (μM)	Limit of Detection (LOD) μM	Comments	References
Dioxetane chemiluminescent probe	Chemiluminescence	0–30	0.91	-	[46]
4APB-modified electrode	Electrochemistry (Cyclic voltammetry)	5–30,000	0.3	Chemical interaction between F^−^ and boronic acid to form a fluorophenylboronate complex.	[47]
Self-assembled monolayer of 4 MPBA	Electrochemistry (EIS)	-	200	F^−^-induced boronic acid conformational change.	[13]
C dots/AuNPs	Fluorimetry	9–117	1.5	3-APBA-derived carbon dots fluorescence FRET behavior to AuNPs was applied.	[48]
Chitosan Cdots	Fluorimetry	6.6–56.6	6.6	Microwave irradiation-derived Cdots, further capped by β-CDs and Calix[4]arene.	[49]
Pyrene boronic acid fluorescent molecule/Cdots	Fluorimetry	0–200	59	The exceptional affinity of F^−^ for the boron atom was deployed for a fluorescent on–off system.	[50]
N-Cdots/morin-Al^3+^	Fluorimetry	0.5–150	0.0558	F^−^ could weaken the IFE between N-doped Cdots and the morin–Al^3+^ system.	[51]
APBA-DSP@AuNPs	Colorimetry	75–1000 and 1000–3000	56.5	AuNPs prior functionalized with DSP, after which 3-APBA was conjugated. Addition of F^−^ resulted in probe aggregation.	[18]
TA-AgNPs	Colorimetry	4–100	0.19	F^−^-induced anti-aggregation action of TA-AgNPs/Al^3+^.	[19]
PAA-AuNPs	Colorimetry	30–200	18	F^−^-induced anti-aggregation effect on the PAA-AuNPs/Al^3+^ system.	[20]
QT-AuNPs/Al^3+^	Colorimetry	25–250 and 250–600	7.5	F^−^-based anti-aggregation action on QT-AuNPs in the presence of Al^3+^.	This work

4-APB = 4-aminophenyl boronic acid; EIS = electrochemical impedance spectroscopy; AuNPs = gold nanoparticles; 3-APBA = 3 aminophenyl boronic acid; Cdots = carbon dots; CDs = cyclodextrins; IFE = inner filter effect; DSP = dithio bis (succinimidylpropionate); PAA = polyacrylic acid; TA = tannic acid; and AgNPs = silver nanoparticles.

**Table 2:** F^−^ detection in tap water, toothpaste, and fluoride mouthwash.

Added Concentration (µM)	Found Concentration (µM)	% Recovery	% RSD (n = 3)
Tap Water			
0	ND	-	-
100	98.93 ± 0.35	98.93	0.48
400	402.5 ± 0.48	100.63	0.55
Toothpaste			
0	71.31 ± 0.95	-	0.88
100	173.48 ± 1.85	102.17	0.94
400	468.93 ± 2.23	99.41	1.14
Fluoride Mouthwash			
0	397.78 ± 1.25	-	1.15
50	446.25 ± 0.91	96.94	1.30
200	595.25 ± 1.41	98.74	1.42

ND = Not detected.

## Data Availability

Data are available within the article and Supplementary Materials.

## Supplementary Materials

The following supporting information can be downloaded at: https://www.mdpi.com/article/10.3390/nano14231967/s1, Figure S1: Particle size distribution of QT-AuNPs, obtained from ImageJ software; Figure S2: UV–Vis absorption spectra of QT-AuNPs monitored over 84 days when stored at 4 °C; Figure S3: UV–Vis absorption spectra of QT-AuNPs monitored for 18 min after the injection of Al^3+^ at 150 µM; Figure S4: Plot of (A_650_/A_520_) against time (min) on QT-AuNPs, charged with Al^3+^ at 150 µM; Figure S5: FTIR spectrum of QT-AuNPs charged with Al^3+^ at 150 µM.
