# Supplementary material for: Quercetin Reduced and Stabilized Gold Nanoparticle/Al3+: A Rapid, Sensitive Optical Detection Nanoplatform for Fluoride Ion
Source: Nanomaterials (Basel). 2024 Dec 7;14(23):1967. doi: 10.3390/nano14231967 (PMC11643675; doi:10.3390/nano14231967)
Supplement: Supplementary file 1 [file nanomaterials-14-01967-s001.zip › nanomaterials-3317700-supplementary.pdf]

nanomaterials

Article

Quercetin reduced and stabilized gold nanoparticle/Al^{3+}: A rapid, sensitive optical detection nanoplatform for Fluoride ion

Titilope John Jayeoye 1,*, Roselina Panghiyangani 2, Sudarshan Singh 3,4 and Nongnuj Muangsin 1,*

[1] Department of Chemistry, Faculty of Science, Chulalongkorn University, Bangkok 10330, Thailand
[2] Department of Biomedic, Faculty of Medicine, Universitas Lambung Mangkurat, Kota Banjarmasin 70123, Indonesia; rpanghiyangani@ulm.ac.id
[3] Faculty of Pharmacy, Chiang Mai University, Chiang Mai 50200, Thailand; sudarshan.s@cmu.ac.th
[4] Office of Research Administration, Chiang Mai University, Chiang Mai 50200, Thailand
* Correspondence: titilope12@gmail.com or jayeoye.t@chula.ac.th (T.J.J.); nongnuj.j@chula.ac.th or nongnuj.ms@gmail.com (N.M.)

Citation: Jayeoye, T.J.; Panghiyangani, R.; Singh, S.; Muangsin, N. Quercetin Reduced and Stabilized Gold Nanoparticle/Al^{3+}: A Rapid, Sensitive Optical Detection Nanoplatform for Fluoride Ion. *Nanomaterials* **2024**, *14*, 1967. https://doi.org/10.3390/nano14231967

Academic Editors: Thomas Pons and Weiping Cai

Received: 31 October 2024
Revised: 5 December 2024
Accepted: 6 December 2024
Published: 7 December 2024

Copyright: © 2024 by the authors. Submitted for possible open access publication under the terms and conditions of the Creative Commons Attribution (CC BY) license (https://creativecommons.org/licenses/by/4.0/).

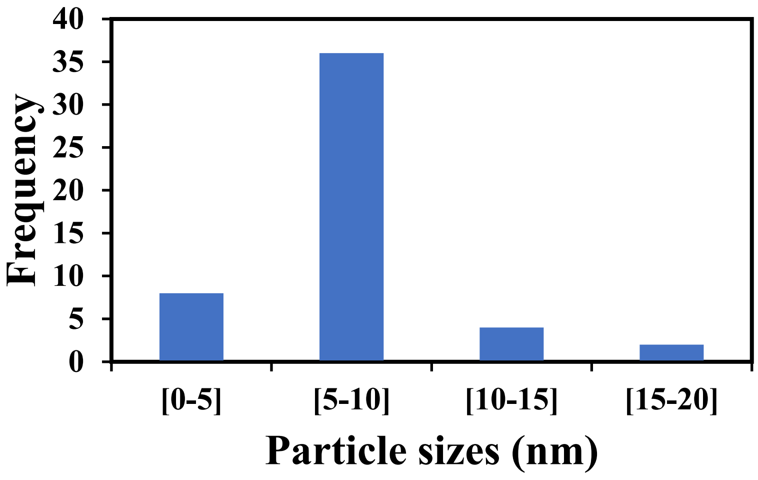

Figure S1. Particle sizes distribution of QT-AuNPs, obtained from Inage J software.

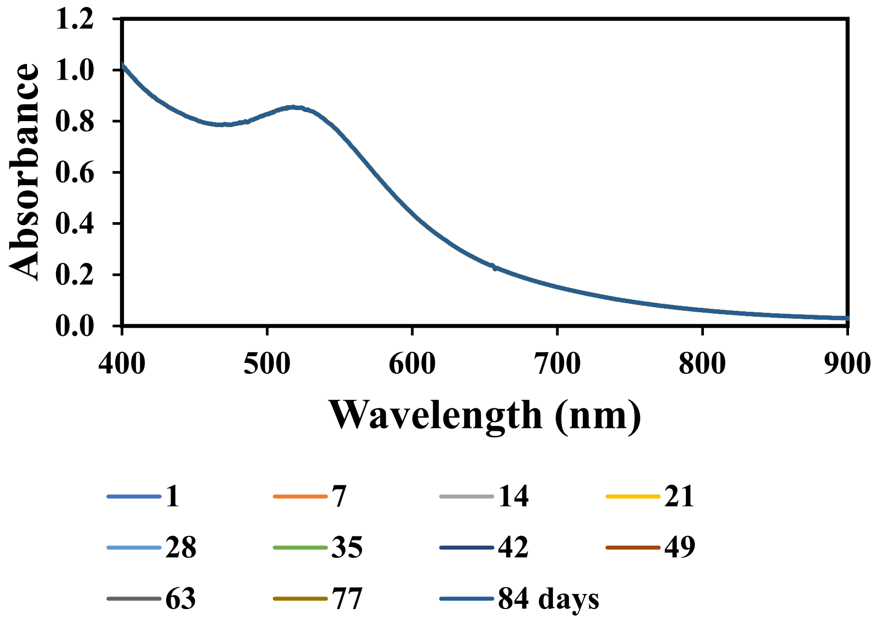

Figure S2. UV-Vis absorption spectra of QT-AuNPs monitored over 84 days, when stored at 4 ^{0}C

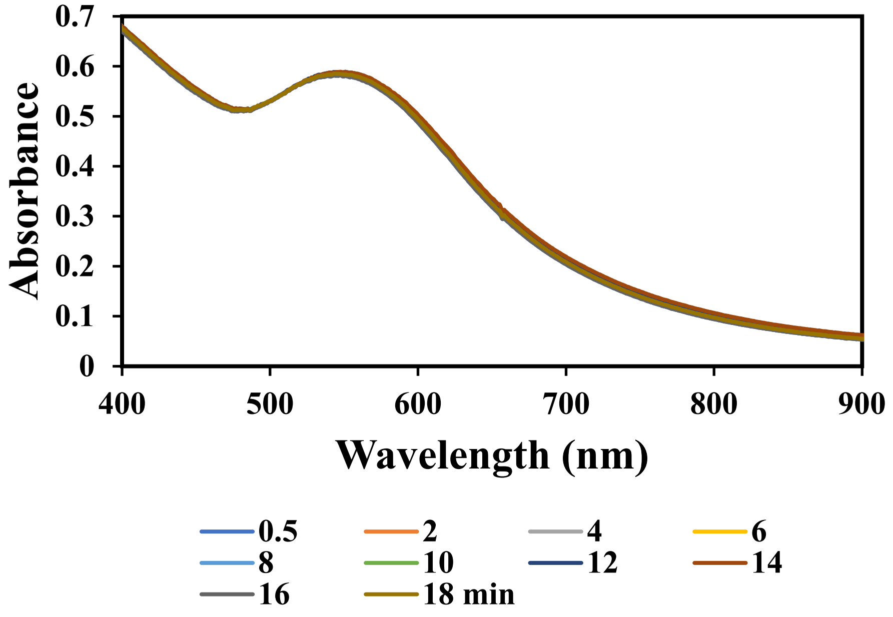

Figure S3. UV-Vis absorption spectra of QT-AuNPs monitored for 18 min after the injection of Al^{3+} at 150 µM.

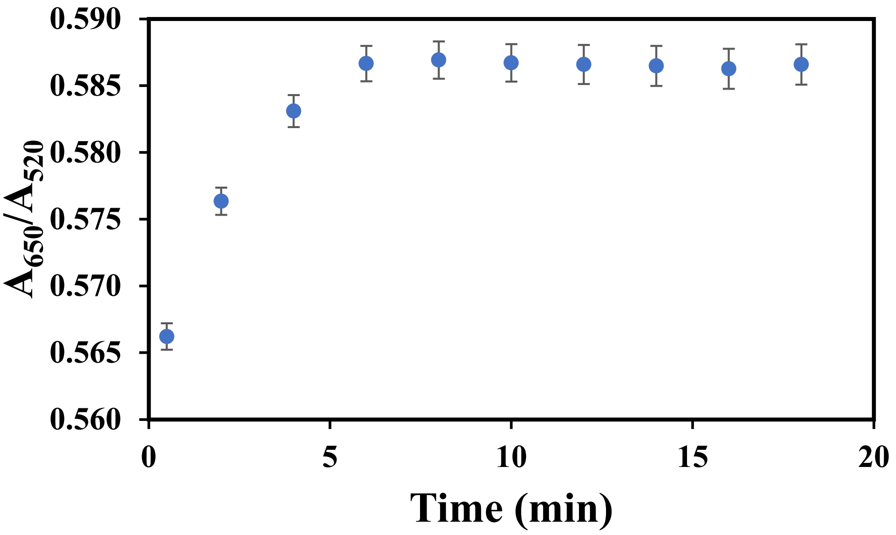

Figure S4. Plot of (A_{650}/A_{520}) against time (min) on QT-AuNPs, charged with Al^{3+} at 150 µM.

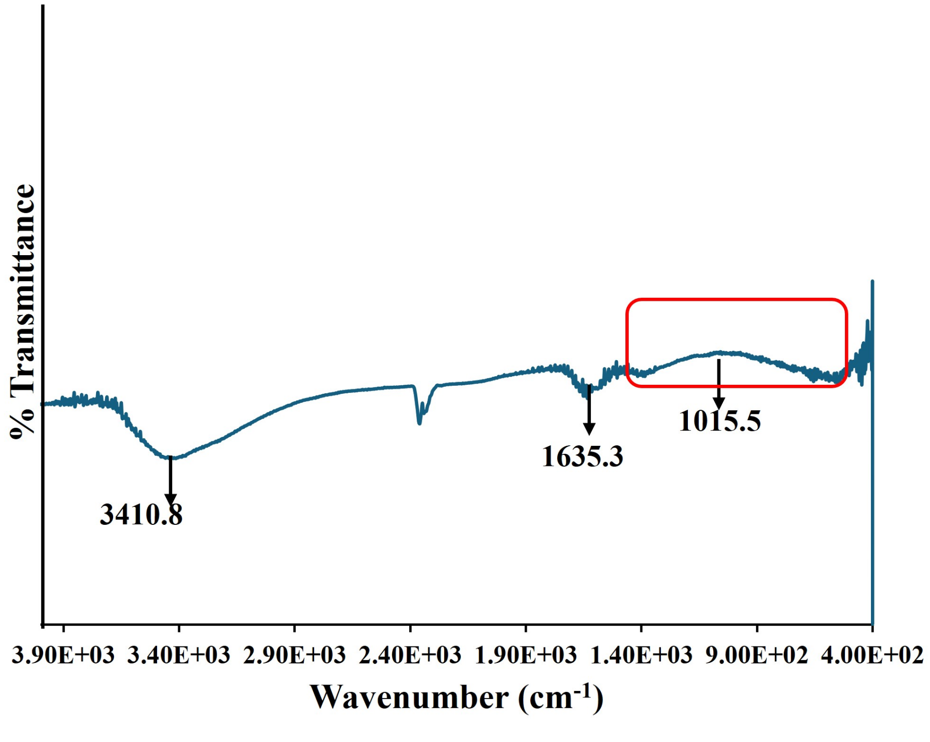

Figure S5. FTIR Spectrum of QT-AuNPs, charged with Al^{3+} at 150 µM.
